# Neither Martyrs nor Clock-Punchers: Rethinking Medical Professionalism in the Age of Burnout

**DOI:** 10.7759/cureus.101066

**Published:** 2026-01-08

**Authors:** Gian Marco Rizzuti

**Affiliations:** 1 Orthopaedics and Traumatology, St. Christophorus Klinik, Münster, DEU

**Keywords:** burnout prevention, health systems, medical education, medical professionalism, physician workforce, surgical training, work life balance

## Abstract

The medical profession is currently facing a profound cultural and structural crisis, often framed as a generational conflict between vocation and work-life balance. Younger physicians are frequently portrayed as less committed, while senior colleagues are associated with an unsustainable culture of sacrifice. This editorial argues that such a dichotomy is misleading and obscures the real drivers of professional dissatisfaction and attrition.

Drawing on recent empirical evidence and real-world observations, the article explores how inadequate training structures, limited supervised exposure, and insufficient institutional support, rather than reduced willingness to work, are eroding professional identity, particularly in surgical disciplines. Burnout is discussed not as an individual failing or primary cause, but as a predictable outcome of systems that combine high responsibility with insufficient preparation and support.

The editorial calls for a renewed professional compact in medicine: one that preserves ethical responsibility and meaningful clinical presence while respecting human limits. By reframing the debate away from moral judgment and toward structural reform, this perspective aims to contribute to a more sustainable and competent medical workforce.

## Editorial

For decades, medicine has been framed as a calling rather than a job. Long hours, personal sacrifice, and unconditional availability were considered defining virtues of the profession. Entire generations of physicians accepted being constantly on call, convinced that endurance was synonymous with professionalism.

Today, this paradigm is increasingly questioned. Younger physicians across Europe and North America are asking for work-life balance, protected working hours, and psychological sustainability. This generational shift is often portrayed as a moral conflict: vocation versus entitlement, dedication versus disengagement.

This framing is misleading and ultimately harmful.

Emerging empirical evidence suggests that the current crisis in medicine, particularly in surgical disciplines, is not driven by a refusal to work hard but by a profound mismatch between responsibility, training, and institutional support.

Evidence from the field: what young surgeons actually say

One of the most comprehensive real-world analyses of this phenomenon is provided by Molteni et al., who investigated the professional experience of surgical residents and surgeons under the age of 45 in Italy through multiple national surveys. Importantly, the surveys include both surgery residents and early-career practicing surgeons, allowing insight into the critical transition from training to independent clinical practice [1].

Their findings challenge many of the prevailing assumptions about younger physicians and professional commitment.

While 85.1% of surgical residents reported working beyond the expected 34 hours per week, only 35% of respondents identified excessive workload as a primary reason for considering abandoning surgery. In contrast, nearly 60% reported having considered dropping out due to an insufficient number of surgical procedures performed, and 87.7% stated that they did not feel adequately prepared for real-world surgical demands. These data indicate that dissatisfaction is driven less by working hours and more by the lack of meaningful clinical exposure and progressive responsibility [1].

Although Molteni et al. focus on the Italian surgical training system, similar patterns of professional dissatisfaction, reduced perceived preparedness, and workforce attrition have been described across different healthcare systems. This suggests that the observed dynamics are not country-specific, but reflect broader structural challenges affecting medical training and early-career practice internationally. 

Far from rejecting effort or sacrifice, young surgeons appear to be rejecting training pathways that demand endurance without delivering competence.

Training without exposure: a silent erosion of professional identity

The disconnect between presence and training is increasingly visible in daily hospital practice. A recent narrative published in the Italian press described senior physicians staffing university hospital wards during major holidays without residents present, raising concerns not about labor rights, but about the erosion of shared clinical responsibility and formative exposure during critical moments of care [2].

Molteni et al. provide the empirical counterpart to this narrative. Although Italian general surgery residents are legally required to participate in hundreds of surgical procedures over the course of their training, survey data suggest that these thresholds are frequently unmet and poorly monitored.

Among practicing surgeons under 45 years of age, the consequences of this gap are evident: only 5% perform more than 50 major surgical procedures per year; only one-third report full autonomy in managing surgical emergencies; approximately 40% report insufficient professional satisfaction [1].

This represents not a failure of motivation, but a failure of training structures. Professional identity in surgery is built through supervised exposure, gradual autonomy, and shared responsibility. When these elements are missing, both confidence and competence erode-often silently.

Burnout is not the root cause: it is the outcome

Burnout is frequently discussed as an individual problem, emphasizing resilience, coping strategies, or generational fragility. However, evidence from the medical education and patient safety literature suggests that burnout is more accurately understood as a systemic outcome rather than a primary cause [3]. This interpretation is consistent with the literature, which frames burnout as a predictable consequence of system design, workload structure, and organizational culture rather than an individual failure of resilience or vocation [3].

More than 80% of surgeons surveyed by Molteni et al. reported experiencing at least one adverse clinical event, yet only 25.8% were familiar with the concept of the “second victim”, despite extensive literature describing its psychological impact on healthcare professionals [1]. The “second victim” phenomenon has been well-described as a predictable response to adverse events in environments characterized by high responsibility and insufficient institutional support [4].

Younger surgeons, in particular, reported behavioral consequences following adverse events, including voluntary withdrawal from complex cases and, in some instances, abandonment of surgical activity. These reactions mirror previously described recovery trajectories among healthcare professionals exposed to medical error without structured support systems [4].

These findings align with broader international observations. In a recent policy-oriented analysis, younger physicians questioned a work culture that equates professionalism with limitless availability, while senior colleagues often interpreted this shift as a loss of vocation rather than a response to structural strain [5].

Burnout, in this context, is not the origin of disengagement but its predictable endpoint.

The false dichotomy: vocation versus sustainability

Public and professional discourse often frames the current crisis as a binary choice: either medicine remains a vocation sustained by personal sacrifice, or it becomes a regulated occupation stripped of ethical depth. The available evidence does not support this dichotomy.

As demonstrated by Molteni et al., younger physicians are not rejecting commitment or responsibility. Instead, they are rejecting empty endurance: long hours without learning, accountability without autonomy, and moral expectations unsupported by functional training systems.

Medicine cannot be reduced to shift work, but neither can it survive on sacrifice alone. When compliance replaces mentorship and presence is eliminated rather than structured, the profession risks losing both its humanity and its competence.

Toward a new professional compact

At the same time, it is essential to acknowledge the systemic pressures faced by senior physicians and healthcare institutions, including staffing shortages, regulatory constraints, and increasing administrative burden, which significantly shape training environments and supervision models [5].

What emerges from these data is the need for a renewed professional compact--one that preserves the ethical core of medicine while acknowledging human limits.

Such a compact should include: realistic enforcement of working-hour regulations paired with protected, high-quality clinical exposure; structured, supervised autonomy, particularly in emergency and high-responsibility settings; institutional recognition and management of the “second victim” phenomenon; evaluation of training quality based on competence and progression rather than endurance alone.

The question is no longer whether medicine is a calling or a job. The question is whether healthcare systems are willing to design environments in which they can responsibly remain both.

If they fail, the loss will not be generational. It will be professional.
